# Supplementary material for: Breeding Maize Hybrids with Improved Drought Tolerance Using Genetic Transformation
Source: Int J Mol Sci. 2024 Oct 2;25(19):10630. doi: 10.3390/ijms251910630 (PMC11477287; doi:10.3390/ijms251910630)
Supplement: Supplementary file 1 [file ijms-25-10630-s001.zip › ijms-3232340-supplementary.pdf]

Table S1. Segregation ratio of *betA* in T1 transgenic plants

Transgenic lines	Origin	No.of $betA^+$	No. of $betA^-$	Expected ratio	χ^2	P
DH4866-003	T0 self-pollinated	22	10	3:1	0.375	P>0.5
DH4866-005	T0 self-pollinated	27	3	15:1	0.22	P>0.5
DH4866-012	T0 backcross	17	13	1:1	0.3	P>0.5
DH4866-042	T0 self-pollinated	26	8	3:1	0.00	P>0.75
DH4866-051	T0 self-pollinated	21	9	3:1	0.18	P>0.5
DH4866-109	T0 self-pollinated	28	4	15:1	1.2	P>0.25
DH4866-120	T0 backcross	13	19	1:1	0.78	P>0.25
DH4866-122	T0 self-pollinated	26	5	15:1	3.62	P>0.05
DH4866-208	T0 backcross	12	17	1:1	0.55	P>0.25
DH4866-213	T0 self-pollinated	20	10	3:1	0.71	P>0.25
DH4866-248	T0 self-pollinated	21	9	3:1	0.18	P>0.5
Qi319-007	T0 self-pollinated	25	7	3:1	0.04	P>0.75
Qi319-032	T0 self-pollinated	24	6	3:1	0.18	P>0.5
Qi319-035	T0 self-pollinated	26	5	3:1	0.87	P>0.25
Qi319-041	T0 self-pollinated	23	7	3:1	0.00	P>0.75
Qi319-087	T0 self-pollinated	25	7	3:1	0.04	P>0.75
Qi319-101	T0 self-pollinated	24	6	3:1	0.18	P>0.5
Qi319-103	T0 self-pollinated	28	2	15:1	0.08	P>0.75
Qi319-124	T0 self-pollinated	21	9	3:1	0.18	P>0.5

T1 plants were examined by PCR for *betA*. When freedom was 1, $\chi^2$0.05=3.84; $\chi^2$0.10=2.71; $\chi^2$0.25=1.32; $\chi^2$0.50 =0.45; $\chi^2$0.75=0.10.

Table S2. Segregation ratio of *betA* in transgenic T2 plants

Lines	Origin	No. of *betA*$^+$	No. of *betA*$^-$	Expected ratio	χ^2	P
DH4866-0031	T0/T1 self-pollinated	30	9	3:1	0.00	P>0.75
DH4866-0032	T0/T1 self-pollinated	24	6	3:1	0.18	P>0.5
DH4866-0033	T0/T1 self-pollinated	26	8	3:1	0.00	P>0.75
DH4866-0121	T0 backcross/T1 self-pollinated	14	17	1:1	0.13	P>0.5
DH4866-0122	T0 backcross /T1 self-pollinated	24	6	3:1	0.18	P>0.5
DH4866-0123	T0 backcross /T1 self-pollinated	23	8	3:1	0.01	P>0.75
DH4866-0421	T0/T1 self-pollinated	21	9	3:1	0.18	P>0.5
DH4866-0422	T0/T1 self-pollinated	26	8	3:1	0.00	P>0.75
DH4866-0423	T0/T1 self-pollinated	20	6	3:1	0.00	P>0.75
DH4866-0511	T0/T1 self-pollinated	22	8	3:1	0.00	P>0.75
DH4866-0512	T0/T1 self-pollinated	21	10	3:1	0.53	P>0.25
DH4866-0513	T0/T1 self-pollinated	30	0	30:0	0.13	P>0.5
DH4866-1091	T0/T1 self-pollinated	30	0	30:0	0.01	P>0.75
DH4866-1092	T0/T1 self-pollinated	29	3	15:1	0.13	P>0.5
DH4866-1093	T0/T1 self-pollinated	28	2	15:1	0.08	P>0.75
DH4866-2081	T0 backcross /T1 self-pollinated	25	7	3:1	0.04	P>0.75
DH4866-2082	T0 backcross /T1 self-pollinated	17	14	1:1	0.13	P>0.5
DH4866-2083	T0 backcross /T1 self-pollinated	15	16	1:1	0.00	P>0.75
Qi319-0321	T0/T1 self-pollinated	20	10	3:1	0.71	P>0.25
Qi319-0322	T0/T1 self-pollinated	22	8	3:1	0.00	P>0.75
Qi319-0323	T0/T1 self-pollinated	23	9	3:1	0.04	P>0.75
Qi319-0411	T0/T1 self-pollinated	24	6	3:1	0.18	P>0.5
Qi319-0412	T0/T1 self-pollinated	21	9	3:1	0.18	P>0.5
Qi319-0413	T0/T1 self-pollinated	25	7	3:1	0.04	P>0.75
Qi319-0871	T0/T1 self-pollinated	31	1	30:0	0.13	P>0.5
Qi319-0872	T0/T1 self-pollinated	30	0	30:0	0.01	P>0.75
Qi319-0873	T0/T1 self-pollinated	24	6	3:1	0.18	P>0.5

T2 plants were examined by PCR for *betA*. When freedom was 1, χ^2 0.05=3.84; χ^2 0.10=2.71; χ^2 0.25=1.32; χ^2 0.50 =0.45; χ^2 0.75=0.10.

Table S3. Betaine concentration of leaves and seeds from WT and transgenic maize plants

Items			NT	DH4866-003111	DH4866-012311	DH4866-042311	DH4866-051211	DH4866-109111
Seeds		Betaine	2.12 ±0.10	6.80±0.18**	7.06±0.34**	8.32± 0.26**	7.10±0.53**	3.70± 0.09**
		CK%	-	321.42%	333.39%	393.23%	335.51%	174.65%

Items			NT	DH4866-003111	DH4866-012311	DH4866-042311	DH4866-051211	DH4866-109111
Leaves	Before stress	Betaine	1.41±0.09f	5.12±0.33e	5.42±0.89e	5.99±0.43de	5.84 ±0.13e	2.59±0.16f
		CK%	-	362.82%	383.82%	424.16%	413.78%	183.30%
	After stress	Betaine	2.23 ±0.25f	7.65±0.51cd	7.91±0.36bc	11.17±1.35a	9.56±0.49ab	5.43±0.93e
		CK%	-	343.05%	354.56%	500.90%	428.85%	243.35%

Lines, growth conditions and statistical analysis were same as described in **Figure 3**. Values represent the mean of three replicates ± SD. Different letters denote statistical significance with $p < 0.05$ using ANOVA and Tukey's HSD test.

Table S4. Yield traits of transgenic lines under drought stress treatment in heading stage for 15 d

Lines	Length of ears (cm)	Rows on ears	No. of kernels /row	100-Kernel weight (g)	Yield/plant (g)	Yield/plot (Kg)	Yield increment (%)
NT (DH4866)	12.21±0.98	12.40±0.89	25.80±1.48	223.04±8.09	140.12±2.56	12.61±0.23	0.00%
DH4866-003111	14.72±0.86**	12.80±1.10	30.40±3.51*	281.52±6.08**	153.06±4.23**	14.08±0.39**	11.66%
DH4866-012211	14.44±0.77**	13.20±1.10	32.20±1.48**	269.55±7.13**	158.08±6.03**	14.54±0.55**	15.33%
DH4866-012311	14.46±0.81**	13.20±1.10	29.80±2.59*	277.02±8.81**	168.55±4.01**	15.51±0.37**	22.96%
DH4866-042111	14.71±0.63**	13.20±1.10	27.80±0.84*	297.82±6.74**	165.01±6.46**	15.18±0.59**	20.38%
DH4866-042311	15.58±0.76**	14.00±0.00*	29.60±1.14**	305.85±7.59**	172.94±7.54**	15.91±0.69**	26.17%
DH4866-051211	15.30±0.71**	13.60±0.89	29.20±2.17*	293.73±8.70**	168.34±5.83**	15.49±0.54**	22.81%
DH4866-051311	15.29±0.97**	13.20±1.10	28.40±1.67*	292.56±7.74**	167.87±4.12**	15.44±0.38**	22.47%
DH4866-109111	14.45±0.54**	13.60±0.89	27.80±3.03	255.76±8.35**	148.72±8.33**	13.68±0.77**	8.50%
DH4866-148211	15.04±0.36**	13.60±0.89	27.60±2.07*	277.96±6.62**	159.60±3.88**	14.68±0.36**	16.43%

Lines, growth conditions and statistical analysis were the same as described in **Figure 5C** and **5D**. The field experiment was carried out in the experiment field under a rain exclusion shelter. Thirty of ears were randomly sampled, and the number of kernel rows and kernel number of each row were counted. The dry kernels were weighed, and mean kernel weight was calculated. Values represent the mean of three replicates ± SD.

Table S5. Yield traits of transgenic hybrids drought-stressed in heading period for 15 d

Lines	Length of ears (cm)	No. of rows on ears	No. of kernels of a row	100-Kernel weight (g)	Yield of a plant (g)	Yield in a plot (Kg)	Yield increment (%)
NT (DH4866×196)	16.21±1.06	15.60±0.89	32.00±2.24	296.88±4.76	164.52±4.64	15.14±0.43	0.00%
DH4866-003111×196	17.92±1.46	16.00±0.00	35.40±2.07*	349.52±3.24**	184.76±4.97**	17.00±0.46**	12.30%
DH4866-003311×196	18.03±1.31	15.60±0.89	35.40±3.13	358.47±5.12**	185.61±11.10**	17.08±1.02**	12.82%
DH4866-012111×196	18.45±1.39*	16.00±0.00	33.60±2.70	371.28±6.02**	189.43±5.26**	17.43±0.48**	15.14%
DH4866-012211×196	17.60±1.40	16.00±0.00	33.20±1.30	367.55±2.76**	196.08±11.67**	18.04±1.07**	19.19%
DH4866-012311×196	18.46±0.93**	16.00±0.00	33.60±1.14	374.02±2.71**	198.55±3.75**	18.27±0.34**	20.68%
DH4866-042111×196	20.18±1.25**	16.40±0.89	34.60±1.67*	343.82±3.10**	205.01±14.93**	18.86±1.37**	24.61%
DH4866-042311×196	20.18±1.52**	16.40±0.89	35.00±1.22*	337.85±1.66**	206.54±17.79**	19.00±1.64**	25.54%
DH4866-051211×196	18.30±0.73**	16.00±0.00	34.40±1.52	337.73±1.54**	197.94±16.52**	18.12±1.52**	19.83%
DH4866-051311×196	18.89±1.57*	16.00±0.00	34.00±2.00	338.35±0.79**	195.87±20.73**	18.02±1.91**	19.06%
DH4866-148111×196	19.33±1.11**	16.00±0.00	33.20±2.28	335.76±2.27**	186.72±2.20**	17.18±0.20**	13.50%
DH4866-148211×196	18.64±0.82**	15.60±0.89	32.50±1.91	326.76±6.05**	183.60±19.37*	16.89±1.78*	11.60%

Lines, growth conditions and statistical analysis were the same as described in **Figure 6C**. Male parent was inbred line 196. The field experiment was carried out in the experiment field under a rain exclusion shelter. Thirty of ears were randomly sampled, and the number of kernel rows and kernel number of each row were counted. The dry kernels were weighed, and mean kernel weight was calculated. Values represent the mean of three replicates ± SD.

Table S6. Yields comparison between transgenic hybrids and non-transgenic controls under both normal growth condition and drought stress in heading period

Hybrids	Normal conditions			Drought stress		
	Yield (kg/ha)	Yield increment (to CK1)	Yield increment (to CK2)	Yield (kg/ha)	Yield increment (to CK1)	Yield increment (to CK2)
Denghai 9# (CK1)	8041.73±581.75ab	0.00%	-	6613.73±488.25c	0.00%	-
Zheng958 (CK2)	8105.30±419.50ab	-	0.00%	7603.47±600.45bc	-	0.00%
DH4866-012311×196	8952.93±436.00a	11.33%	10.46%	8731.03±266.52ab	32.02%	14.83%
DH4866-042311×196	9180.20±304.14a	14.16%	13.26%	8931.83±152.22a	35.03%	17.46%
DH4866-051211×196a	9003.60±346.24a	11.96%	11.08%	8804.33±346.27ab	33.13%	15.79%
Qi319-032111×Chang7-2	8979.73±181.61ab	11.66%	10.79%	8443.17±404.26ab	27.67%	11.04%
Qi319-032211×Chang7-2	8836.33±369.90ab	9.88%	9.02%	8631.20±300.65ab	30.51%	13.52%
Qi319-041211×Chang7-2	8707.90±343.90ab	8.28%	7.43%	8273.47±599.85ab	25.10%	8.81%
DH4866-042311×340	9061.33±360.42a	12.68%	11.80%	8601.60±400.20ab	30.07%	13.13%
Luyuan92× Qi319-032111	8668.77±385.58ab	7.80%	6.95%	8572.50±360.47ab	29.63%	12.74%

Lines, growth conditions and statistical analysis were the same as described in **Figure 6D**. Transgenic hybrids (F1) and 2 commercial hybrids in China, Zheng958 and Denghai 9# were sown in plots under normal growth conditions and gave water deficit by without irrigation at heading period. The number of plants, the number and weight of ear were collected at harvest, respectively. 30 of ears were randomly sampled, and the number of kernel rows and kernel number of each row were counted. The dry kernels were weighed, and mean kernel weight was calculated. Values represent the mean of three replicates ± SD. Different letters denote statistical significance with $p < 0.05$ using ANOVA and Tukey's HSD test.
